# Risk of herpes zoster in psoriasis patients receiving systemic therapies: a nationwide population-based cohort study

**DOI:** 10.1038/s41598-021-91356-3

**Published:** 2021-06-03

**Authors:** Sze-Wen Ting, Sze-Ya Ting, Yu-Sheng Lin, Ming-Shyan Lin, George Kuo

**Affiliations:** 1 Department of Dermatology, New Taipei City Tu-Cheng Municipal Hospital, New Taipei City, Taiwan; 2 Department of Pediatric Surgery, Linkou Chang Gung Memorial Hospital, Taoyuan City, Taiwan; 3 Department of Cardiology, Chiayi Chang Gung Memorial Hospital, Chiayi County, Taiwan; 4 Chang Gung University, Taoyuan City, Taiwan; 5 Department of Nephrology, Kidney Research Center, Linkou Chang Gung Memorial Hospital, No. 5, Fuxing Street, Guishan District, Taoyuan City, 33305 Taiwan

**Keywords:** Infectious diseases, Skin diseases, Epidemiology

## Abstract

The incidence of herpes zoster in psoriasis patients is higher than in the general population. However, the association between herpes zoster risk and different systemic therapies, especially biologic agents, remains controversial. This study investigated the association between herpes zoster risk and several systemic antipsoriasis therapies. This prospective open cohort study was conducted using retrospectively collected data from the Taiwan National Health Insurance Research Database. We included 92,374 patients with newly diagnosed psoriasis between January 1, 2001, and December 31, 2013. The exposure of interest was the “on-treatment” effect of systemic antipsoriasis therapies documented by each person-quarter. The outcome was the occurrence of newly diagnosed herpes zoster. During a mean follow-up of 6.8 years, 4834 (5.2%) patients were diagnosed with herpes zoster after the index date. Among the systemic antipsoriasis therapies, etanercept (hazard ratio [HR] 4.78, 95% confidence interval [CI] 1.51–15.17), adalimumab (HR 5.52, 95% CI 1.72–17.71), and methotrexate plus azathioprine (HR 4.17, 95% CI 1.78–9.82) were significantly associated with an increased risk of herpes zoster. By contrast, phototherapy (HR 0.76, 95% CI 0.60–0.96) and acitretin (HR 0.39, 95% CI 0.24–0.64) were associated with a reduced risk of herpes zoster. Overall, this study identified an association of both etanercept and adalimumab with an increased risk of herpes zoster among psoriasis patients. Acitretin and phototherapy were associated with a reduced risk.

## Introduction

Psoriasis is a chronic inflammatory dermatosis whose pathogenesis involves various immune cells and cytokines. The interplay between plasmacytoid and myeloid dendritic cells, helper T cells (Th1, Th17, Th22), and mediators such as tumor necrosis factor (TNF)-α, interleukin (IL)-12, IL-23, and IL-17 provides targets for psoriasis drug development^1,2^. Several biologic agents have been approved for treating psoriasis, such as anti-TNF-α agents and IL-12/IL-23 antagonists. However, because of the resulting suppression of cell-mediated immunity, addressing the risks of bacterial and viral infections remains of paramount importance^3^.

Herpes zoster (HZ) is caused by the reactivation of varicella–zoster virus (VZV) from the dorsal root ganglia in individuals who have had a previous varicella infection. VZV-specific CD4+ and CD8+ T cells as well as VZV-specific antibodies participate in the immune response during primary infection and reactivation. Notably, a low number of T cells or low antibody titer has been associated with increased severity of HZ and postherpetic neuralgia^4^. Although HZ is usually not life-threatening, it has been associated with increased health care utilization and medical expenditure. The pain caused by HZ impairs the quality of life as well^5^. Tsai et al. reported a higher risk of HZ in psoriasis patients than in the general population. This risk is even higher in patients receiving systemic therapy than in those using topical treatment alone^6^. Research has yet to identify a significantly different risk of HZ between psoriasis patients treated with biologic agents and those receiving other systemic therapies; nevertheless, the suppression of T cell–mediated immunity leads to the reasonable inference that biologic agents may be associated with an increased risk of HZ^7,8^.

To determine the risk of HZ in psoriasis patients receiving various medications, we conducted a nationwide population-based cohort study.

## Material and methods

### Data source

This was a prospective open cohort study that incorporated retrospectively collected data from the Taiwan National Health Insurance Research Database (NHIRD). The NHIRD contains no identifiable personal information, contains data on roughly 99.8% of the 23 million residents of Taiwan, and provides information regarding diagnoses, prescriptions, examinations, surgeries, and expenditures relating to both inpatient and outpatient services since March 1995. Because of the affordability and mandatory enrollment of Taiwan’s National Health Insurance (NHI) program, long-term follow-up is nearly complete. Further information regarding the NHI and NHIRD has been described in previous publications^9–11^. This study was approved by the Institutional Review Board (IRB) of Chang Gung Memorial Hospital and was conducted in accordance with the principles of the Declaration of Helsinki.

### Study population

**Figure 1:**
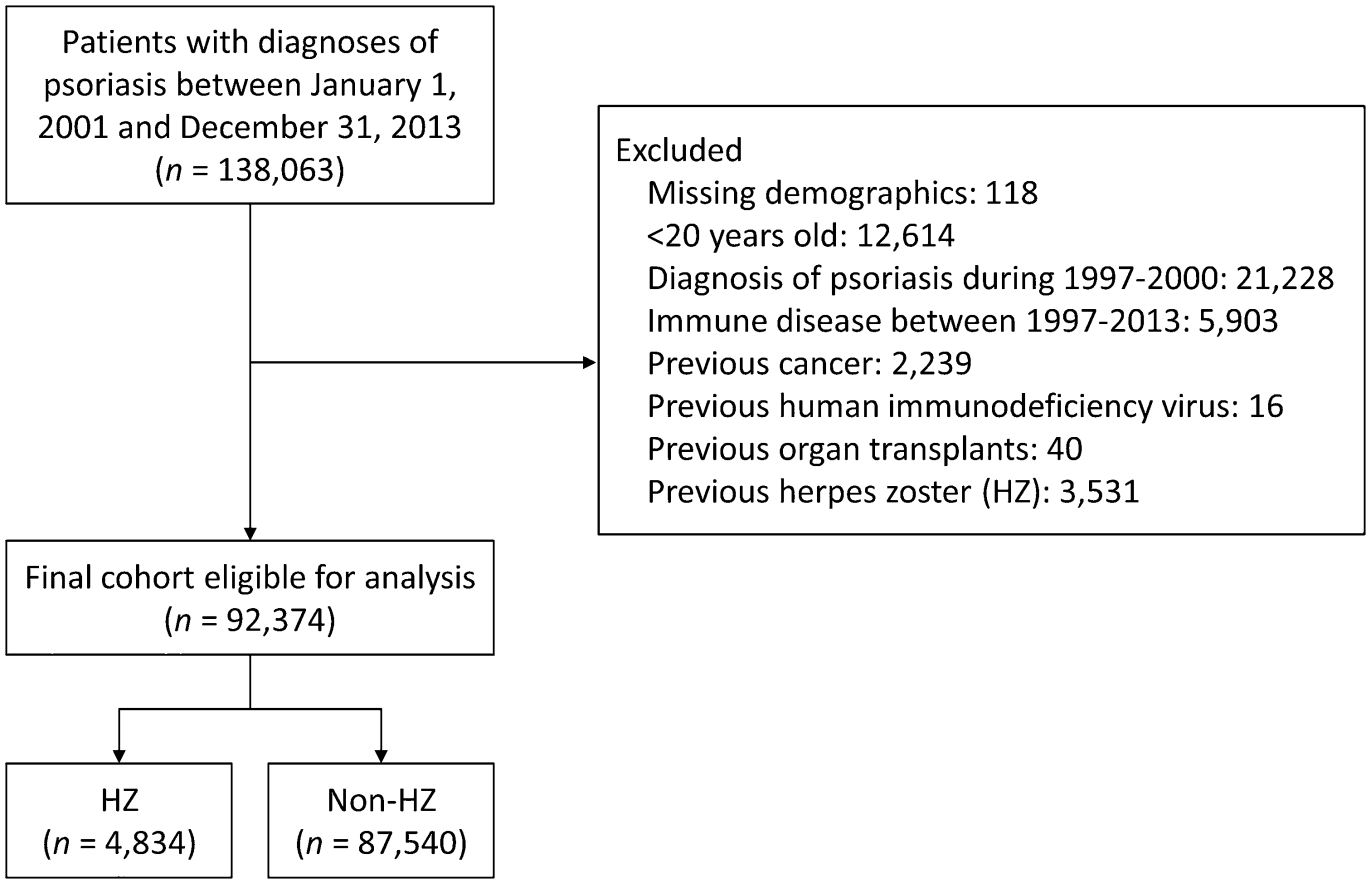
Flowchart of patient inclusion.

In this study, patients of interest were those diagnosed with psoriasis between January 1, 2001, and December 31, 2013. Psoriasis patients were required to have had three outpatient diagnoses or any inpatient diagnosis based on *International Classification of Diseases, Ninth Revision, Clinical Modification* (ICD-9-CM) codes 6960, 6961, and 6968, as documented in the NHIRD^12,13^. Patients who had been diagnosed with psoriasis before 2000 were excluded; hence, the analyzed cohort included only new-onset psoriasis patients. Furthermore, the first psoriasis diagnosis date was defined as the index date. We excluded patients with missing demographics (< 0.1%) and those younger than 20 years old. We additionally excluded the following diseases because they were competing indications for several antipsoriatic biologic agents and immunosuppressants: systemic lupus erythematosus (710.xx), rheumatoid arthritis (714.xx), inflammatory bowel disease (Crohn’s disease [555.xx] and ulcerative colitis [556.xx]), ankylosing spondylitis (720.xx), and hidradenitis suppurativa (705.83). Also excluded were severely immunocompromised patients who had a health status involving treatment with an effect that would overshadow that of antipsoriatic therapy: cancer (140–280), human immunodeficiency virus (HIV) infection (042–044), or any organ transplant (V42.xx). Finally, patients who had any diagnosis of HZ before the index date were also excluded (Fig. 1).

### Unit of follow-up time and definition of exposures

We divided each calendar year into four quarters for each patient and year after psoriasis diagnosis. The analytic unit was 1 person-quarter. Person-quarters were used because medications for chronic illnesses are refilled for a maximum of 3 months according to the Taiwan NHI reimbursement policy^14,15^. The covariates and exposures were assessed for each person-quarter.

The following medications or therapies were of interest in the study: methotrexate (MTX), acitretin, cyclosporine, tacrolimus, mycophenolate mofetil, mycophenolic acid, azathioprine, etanercept, adalimumab, ustekinumab, and ultraviolet (UV) phototherapy. In the quarters without incident HZ, the use of oral drugs was defined to include at least 30 prescription days, this includes MTX, which is counted by “days” in the database. The use of UV phototherapy was defined as at least eight sessions in the quarter.

Because of the retrospective nature of this study, the starting date of each patient may differ. Therefore, some patients may receive biologic treatment across two consecutive quarters, but in each quarter, the prescription duration was only two weeks. Considering the different dosing and prescription of biologic agents, we counted these agents with a slightly different way: Ustekinumab, at least one administration in the quarter; at least on two administration of adalimumab in the quarter; etanercept, at least eight administrations in the quarter. If the treatment schedule spanned over two quarters, we count the use by at least one administration of adalimumab and at least four administration of etanercept within 14 days in the starting or ending quarter. Notably, in the quarters with incident HZ, medications of primary interest were identified before the diagnostic date of HZ in which ustekinumab was tracked back to 3 months, whereas the other drugs (including UV phototherapy) were tracked back to 1 month^7,16,17^.

In addition, we extracted the cumulative prednisone-equivalent dose of oral glucocorticoid during the quarter. Statin use was also extracted during the quarter and was defined as at least 30 prescription days. Information about medications was extracted from the claims data of outpatient visits or pharmacy refills for chronic illness medications by using the Anatomical Therapeutic Chemical codes or the Taiwan NHI reimbursement code.

### Covariates and outcome

The covariates in this study were as follows: age at the index date, sex, urbanization level of place of residence, diabetes mellitus, hypertension, dyslipidemia, chronic kidney disease, gouty arthritis, psoriatic arthritis, and the Charlson comorbidity index score. Comorbidities were defined as having at least two outpatient diagnoses or an inpatient diagnosis in the previous year. The ICD-9-CM diagnostic codes of the comorbidities are listed in the supplement (Supplemental Table 1). The study outcome was incident HZ, which was defined as the presence of ICD-9-CM diagnostic code 053 with at least two outpatient visits within 14 days or one inpatient diagnosis^18–20^. All patients were followed from the index date to either the date of incident HZ, the date of withdrawal from the NHI program, or December 31, 2013, whichever came first.

### Statistical analysis

Data were expressed as frequency and percentage for categorical variables and as mean and standard deviation for continuous variables. Each person-quarter was classified into mutually exclusive exposures or control periods (see Table 2). The incidence of “on-treatment,” namely each control period or exposure period, was calculated by dividing the number of HZ cases by net person-years on treatment. We first compared the risk of incident HZ among the exposure or control periods without adjustment of covariates. Next, the associations between covariates, medications of interest, and HZ risk were investigated using a multivariable time-dependent Cox proportional hazards model. The outcome dependency among the multiple person-quarters of each patient was accounted for by using a robust (sandwich) estimator. A two-sided *P* value of < 0.05 was considered to be statistically significant, and no adjustment of multiple testing (multiplicity) was made. All the statistical analyses were performed using R version 3.6.1 (R Development Core Team) with the ‘*rms*’ package (Version 5.1-3.1 updated on April 22, 2019).

### Ethics approval and consent to participate

The NHIRD contains no identifiable personal information; the need for informed consent was waived by the Institutional Review Board of Chang Gung Memorial Hospital. because of this study’s retrospective noninterventional design and because patient data confidentiality and privacy were maintained. The study was approved by the Institutional Review Board of Chang Gung Memorial Hospital.

## Results

A total of 138,063 psoriasis patients were identified between 2001 and 2013. After excluding patients with missing demographics (n = 118), patients younger than 20 years old (n = 12,614) and patients having an autoimmune disease (n = 5903), cancer (n = 2239), HIV infection (n = 16), organ transplant (n = 40), or previous HZ (n = 3531), the remaining 92,374 new-onset psoriasis patients were eligible for the final analysis. During the entire follow-up, 4834 (5.2%) patients were diagnosed with HZ after the index date (Fig. 1).

Table 1 summarizes the patients’ demographic and clinical characteristics. The average age of the patients at baseline was 46.7 years, with a mean follow-up duration of 6.8 years (standard deviation: 3.7 years). Male patients constituted approximately 60%, and the most frequently used therapies were phototherapy, acitretin, and MTX either at baseline or during the entire follow-up (Table 1).

**Table 1:** Demographic and clinical characteristics of patients at baseline and through follow-up.

Variable	Baseline	Through the follow up
Age (years; mean ± SD)	46.7 ± 17.2	53.5 ± 17.0*
Female sex, n (%)	36,501 (39.5)	–
**Urbanization level of patient’s residence, n (%)**		
Low	7282 (7.9)	–
Moderate	28,426 (30.8)	–
High	28,704 (31.1)	–
Very High	27,962 (30.3)	–
**Comorbidity, n (%)**		
Diabetes mellitus	8577 (9.3)	18,417 (19.9)
Hypertension	17,260 (18.7)	33,196 (35.9)
Dyslipidemia	7818 (8.5)	24,082 (26.1)
Chronic kidney disease	3033 (3.3)	10,821 (11.7)
Gouty arthritis	4703 (5.1)	12,383 (13.4)
Psoriasis arthritis	65 (0.1)	7390 (8.0)
Charlson Comorbidity Index score (mean ± SD)	0.5 ± 1.0	0.9 ± 1.7*
**Medication of primary interest, n (%)**		
PUVA or UVB	3583 (3.9)	12,728 (13.8)
Cyclosporine	274 (0.3)	2201 (2.4)
Oral retinoids (Acitretin)	1770 (1.9)	6496 (7.0)
Tacrolimus	0 (0.0)	41 (0.04)
Hydroxyurea	7 (0.01)	102 (0.1)
Mycophenolate mofetil	0 (0.0)	43 (0.05)
Azathioprine	120 (0.1)	906 (1.0)
Methotrexate without any biological agents	2921 (3.2)	12,301 (13.3)
Etanercept without methotrexate	0 (0.0)	215 (0.2)
Adalimumab without methotrexate	0 (0.0)	187 (0.2)
Ustekinumab without methotrexate	0 (0.0)	129 (0.1)
Methotrexate with any biological agents	0 (0.0)	396 (0.4)
**Steroid use at diagnosis of psoriasis (prednisone-equivalent dose)**		
Never user	79,909 (86.5)	–
< 5 mg/day	11,722 (12.7)	–
≥ 5 mg/day	743 (0.8)	–
Statin	2007 (2.2)	16,771 (18.2)

*SD* standard deviation, *PUVA* photochemotherapy, *UVB* ultraviolet B.

Data are presented as frequency (percentage) or mean ± standard deviation.

*At the end of the follow-up.

The crude incidence rate of HZ was 8.0 events per 1,000 person-years (Table 2). Table 3 lists the results of multivariable time-dependent Cox analysis. For systemic therapy, we found that etanercept (hazard ratio [HR] 4.78, 95% confidence interval [CI] 1.51–15.17), adalimumab (HR 5.52, 95% CI 1.72–17.71), and MTX plus azathioprine (HR 4.17, 95% CI 1.78–9.82) were significantly associated with an increased risk of HZ. The combination of MTX with any biologic agent (etanercept, adalimumab, or ustekinumab) exhibited a non-significant higher risk of HZ (HR 3.10, 95% CI 0.97–9.92, *P* = 0.056). By contrast, phototherapy (HR 0.76, 95% CI 0.60–0.96), acitretin (HR 0.39, 95% CI 0.24–0.64), and MTX plus phototherapy (HR 0.29, 95% CI 0.09–0.90) were associated with lowerrisk of HZ.

**Table 2:** Crude incidence of herpes zoster per 1000 person-years for each treatment.

Variable	No. of person-quarter	Net person-year on treatment	No. of herpes zoster	Incidence (95% CI)*	*P* value versus control period
Control period	2,375,268	576,853.3	4628	8.0 (7.8–8.3)	–
Methotrexate with any biological agents	1781	440.7	3	6.8 (− 0.9 to 14.5)	0.042
Etanercept without Methotrexate	1063	262.9	3	11.4 (− 1.5 to 24.3)	0.008
Adalimumab without Methotrexate	926	229.2	3	13.1 (− 1.7 to 27.9)	0.001
Ustekinumab without Methotrexate	249	61.7	0	NA	NA
Methotrexate with PUVA or UVB	7093	1729.4	3	1.7 (− 0.2 to 3.7)	0.027
Methotrexate with Acitretin	2233	545.5	2	3.7 (− 1.4 to 8.7)	0.368
Methotrexate with Azathioprine	1095	267.9	5	18.7 (2.3–35.0)	0.001
Methotrexate only	48,839	11,799.0	79	6.7 (5.2–8.2)	0.522
Acitretin only	17,484	4173.4	16	3.8 (2.0–5.7)	< 0.001
Acitretin with PUVA or UVB	4591	1113.4	4	3.6 (0.1–7.1)	0.092
PUVA or UVB only	41,144	9824.7	71	7.2 (5.5–8.9)	0.055
Azathioprine only	2262	550.7	7	12.7 (3.3–22.1)	0.060
Other combinations	4521	1101.2	10	9.1 (3.5–14.7)	0.056
Total	2,508,549	608,964.3	4834	7.9 (7.7–8.2)	–

*NA* not applicable, *PUVA* photochemotherapy, *UVB* ultraviolet B.

*Per 1000 person-years.

**Table 3:** Associations between covariates, medications, and risk of herpes zoster.

Variable	HR (95%CI)	*P*
Age, per year*	1.036 (1.03–1.04)	< 0.001
Female sex	1.10 (1.04–1.17)	0.001
**Urbanization level**		
Low	Reference	–
Moderate	1.02 (0.92–1.14)	0.666
High	1.00 (0.89–1.11)	0.939
Very high	1.04 (0.93–1.15)	0.527
Comorbid conditions*		
Diabetes mellitus	0.92 (0.84–1.01)	0.068
Hypertension	1.11 (1.03–1.19)	0.004
Dyslipidemia	1.18 (1.08–1.29)	< 0.001
Chronic kidney disease	0.95 (0.84–1.08)	0.451
Gouty arthritis	0.92 (0.82–1.03)	0.141
Psoriasis arthritis	1.25 (1.01–1.53)	0.035
Charlson Comorbidity Index score*	1.18 (1.15–1.22)	< 0.001
Medication of primary interest*		
Control period	Reference	–
Methotrexate with any biological agents	3.10 (0.97–9.92)	0.056
Etanercept without Methotrexate	4.78 (1.51–15.17)	0.008
Adalimumab without Methotrexate	5.52 (1.72–17.71)	0.004
Ustekinumab without Methotrexate	NA	NA
Methotrexate with PUVA or UVB	0.29 (0.09–0.90)	0.032
Methotrexate with Acitretin	0.58 (0.15–2.32)	0.442
Methotrexate with Azathioprine	4.17 (1.78–9.82)	0.001
Methotrexate only	1.01 (0.80–1.27)	0.958
Acitretin only	0.39 (0.24–0.64)	< 0.001
Acitretin with PUVA or UVB	0.44 (0.17–1.18)	0.104
PUVA or UVB only	0.76 (0.60–0.96)	0.023
Azathioprine only	1.59 (0.76–3.33)	0.218
Other combinations	1.50 (0.78–2.85)	0.222
Medication not of primary interest		
Steroid (prednisone-equivalent dose)*		
Never user	Reference	–
< 5 mg/day	0.99 (0.91–1.08)	0.786
≥ 5 mg/day	1.68 (1.24–2.27)	0.001
Statin*	1.54 (1.38–1.71)	< 0.001

*PUVA* photochemotherapy, *UVB* ultraviolet B, *HR* hazard ratio, *CI* confidence interval, *NA* not applicable.

*Time-varying covariates that may change in each person-quartile.

In addition, older age, female sex, hypertension, dyslipidemia, psoriatic arthritis, and a relatively high Charlson comorbidity index score were observed to be associated with an increased risk of HZ. Finally, concurrent exposure to a steroid at an equivalent dose higher than a 5-mg prednisolone per day (HR 1.68, 95% CI 1.24–2.27) or statin (HR 1.54, 95% CI 1.38–1.71) was associated with higher risk of HZ (Table 3). In the alternative model, each medication was treated as a covariate rather than pre-specified combinations of therapy and the results showed that adalimumab, etanercept and mycophenolate mofetil/mycophenolic acid were associated with higher risks, whereas phototherapy and acitretin were associated with lower risks. (Supplemental Table 2).

## Discussion

This nationwide population-based cohort study yielded real-world evidence of HZ risk in psoriasis patients receiving systemic therapy. We observed that treatment with etanercept, adalimumab, and MTX plus AZA were significantly associated with higher risk of HZ. Furthermore, MTX combined with any biologic agent non-significantly associated with HZ risk. By contrast, acitretin and phototherapy were associated with lower risk of HZ. The patients’ characteristics associated with increased HZ include age, female sex, steroid use, hypertension, dyslipidemia, and psoriatic arthritis.

The risk of HZ was higher in patients with immunocompromised statuses, such as those with HIV, cancer, and allogeneic solid organ or hematopoietic stem cell transplants^21–23^. Associations between HZ and autoimmune diseases, including systemic lupus erythematosus, rheumatoid arthritis, and inflammatory bowel disease, and between immunosuppressant use and HZ have been reported^16,24–27^. Data pooled both from the meta-analysis of clinical trials and from observational studies show that biologic agents against rheumatic disease increased the risk of HZ^28^. However, the association between HZ risk and biologic agents against psoriasis is not clear. For example, Dreiher et al. compared various systemic treatments with control period measurements and found that only steroid use was associated with a higher risk of HZ^29^. Shalom et al. reported an increased risk of HZ in patients receiving a combination of MTX plus biologic agents; however, in a large cohort, they observed no increased risk in patients receiving biologic agents alone^7^. Shalom et al. subsequently demonstrated that therapy with biologic agents and MTX did not entail a higher risk of HZ than the risk in the composite reference group, which included patients treated with phototherapy, oral steroids, topical therapy, and immunomodulators other than MTX (HR 2.22 [95% CI 0.82–5.97; *P* = 0.116] for TNF-α inhibitors, 2.73 [0.98–7.58; *P* = 0.054] for ustekinumab, and 1.04 [0.20–5.41; *P* = 0.966] for MTX)^8^. This is the first large cohort study to demonstrate an increased risk of HZ among psoriasis patients receiving etanercept or adalimumab. We observed no occurrence of HZ after exposure to ustekinumab, which may be attributed to limited exposure time due to the late approval of ustekinumab by the Taiwan Food and Drug Administration in 2011.

In this study, acitretin was associated with reduced risk of HZ, which was similarly reported by Shalom et al. Acitretin is a mediator of cell differentiation and immune function modulation whose use may not be associated with profound immunosuppression. Retinoic acid–inducible gene-I (RIG-I), a pattern recognition receptor that identifies viral RNA transcripts, is upregulated by retinoic acid and its intracellular receptors^30^. RIG-I participates in the immune response against herpes simplex virus and VZV^4,31,32^. Additional evidence has emerged from a study of hematopoietic stem cell transplant patients. High et al. showed that low plasma retinol concentration was associated with increased HZ during the peri-transplantation period^33^. From a clinical perspective, psoriasis patients who achieve disease control with an acitretin-only regimen may experience less immune suppression compared with those receiving stronger immunosuppressive agents.

Next, we observed that UV phototherapy reduced the risk of HZ. UV light exerts immunomodulatory effects such as inducing a shift from Th1- to Th2-mediated response, increasing regulatory T cell function, augmenting macrophage differentiation, and inhibiting plasma cell differentiation^34^. Therefore, UV phototherapy is widely used as a systemic therapy for psoriasis. Although the effect of UV light on the skin is mainly anti-inflammatory, this may not imply an overt immunosuppressive effect. Upon exposure to UV light, the human skin generates vitamin D, a vital nutrient for skeletal health and a well-known immunoregulator. Vitamin D can upregulate several antimicrobial peptides, facilitate macrophage function, and modulate inflammation elicited by T cells and B cells. Although certain immunosuppressive effects of high-dose vitamin D on lymphocytes have been reported in animal and cell studies, vitamin D supplements can be used to enhance T cell–mediated immunity in vitamin D–deficient patients^35^. Chao et al. reported that vitamin D–deficient hemodialysis patients had lower immunity against VZV and that active vitamin D therapy lowered the risk of HZ^36,37^. Patients with psoriasis are also susceptible to vitamin D deficiency, which may be attributable to chronic inflammation and steroid exposure. Moreover, Vahavihu et al. demonstrated an improved vitamin D serum level and cutaneous expression of cathelicidin in patients with concurrent psoriasis and vitamin D deficiency^38^. We contend that the protective effect of UV phototherapy is due to an increased vitamin D level associated with scheduled and carefully monitored UV exposure.

Similar to previous studies on general population or on diabetic patients, the use of statin is associated with higher risk of HZ. The actual mechanisms of these association have not fully understood, but may involve the effect of statin on T cell function^39–43^.

Our study has the strength of being a cohort study that used a time-dependent design to assess the risk of HZ associated with different treatment exposures. This study, however, has limitations. First, ustekinumab was approved and became available in Taiwan in 2011; therefore, the duration of follow-up for this drug was shorter than that of other medications. Second, several biologic agents, including infliximab, efalizumab, and alefacept, are not approved in Taiwan for treating psoriasis. Therefore, the number of patients exposed to biologic agents will be relatively small, which could lead to the risk estimates underpowered. Third, because the NHIRD data did not include psoriasis severity or laboratory findings, we were unable to adjust for these parameters in our model. Previous studies of psoriasis using NHIRD used the presence of systemic therapies or psoriatic arthritis to classify patients into “moderate-to-severe” category as compared to the “mild” category. This classification could reflect long-term morbidities in psoriasis patients^12,13,44–46^. In addition, the changes in disease severity are accompanied with medication adjustment, which could be adjusted in our study because they were time-varying covariates. Therefore, this study design may partially mitigate the lack of severity score.

In conclusion, this study found that patients with moderate to severe psoriasis who were treated with certain biologic agents had a higher risk of HZ, whereas those treated with UV phototherapy or acitretin were associated with lower risk of HZ.

## Supplementary Information

Supplementary Tables.

## Data Availability

The data underlying this study is from the National Health Insurance Research Database (NHIRD), which has been transferred to the Health and Welfare Data Science Center (HWDC). Interested researchers can obtain the data through formal application to the HWDC, Department of Statistics, Ministry of Health and Welfare, Taiwan (http://dep.mohw.gov.tw/DOS/np-2497-113.html).
