# Supplementary material for: Risk of herpes zoster in psoriasis patients receiving systemic therapies: a nationwide population-based cohort study
Source: Sci Rep. 2021 Jun 3;11:11824. doi: 10.1038/s41598-021-91356-3 (PMC8175372; doi:10.1038/s41598-021-91356-3)
Supplement: Supplementary file 1 — Supplementary Tables. [file 41598_2021_91356_MOESM1_ESM.docx]

Supplemental Material To:

Risk of herpes zoster in psoriasis patients receiving systemic therapies: a nationwide population-based cohort study

Running title: anti-psoriasis therapies and herpes zoster

Sze-Wen Ting, MD, Sze-Ya Ting, MD, Yu-Sheng Lin, MD, Ming-Shyan Lin, MD, George Kuo, MD

**Supplemental Table 1**. ICD-9 CM diagnostic codes

| Variable | ICD-9 CM Code |
| --- | --- |
| Psoriasis | 696.xx |
| Psoriasis arthritis | 696.0 |
| Immune disease | 555.xx, 556.xx, 710.xx, 714.xx, 720.xx, 705.83 |
| Malignancy | 140.xx-208.xx (Catastrophic illness card) |
| Human immunodeficiency virus | 042.xx-044.xx |
| Organ transplants | V42.xx |
| Herpes zoster | 053.xx |
| Diabetes mellitus | 250.xx |
| Hypertension | 401.xx-405.xx |
| Dyslipidemia | 272.xx |
| Chronic kidney disease | 580.xx-589.xx, 403.xx-404.xx, 016.0x, 095.4x, 236.9x, 250.4x, 274.1x, 442.1x, 447.3x, 440.1x, 572.4x, 642.1x, 646.2x, 753.1x, 283.11, 403.01, 404.02, 446.21 |
| Gouty arthritis | 274.xx |

ICD-9 CM, International Classification of Diseases, Ninth Revision, Clinical Modification.

**Supplemental Table 2.** Associations between covariates, each medication (rather than combination of medications) and risks of herpes zoster

| Variable | HR (95% CI) | *P* |
| --- | --- | --- |
| Age, per year* | 1.032 (1.030–1.034) | <0.001 |
| Female sex | 1.14 (1.08–1.21) | <0.001 |
| Urbanization level |  |  |
| Low | Reference |  |
| Moderate | 1.07 (0.96–1.19) | 0.216 |
| High | 1.06 (0.95–1.18) | 0.272 |
| Very High | 1.13 (1.02–1.26) | 0.024 |
| Comorbid conditions* |  |  |
| Diabetes mellitus | 0.96 (0.88–1.04) | 0.331 |
| Hypertension | 1.09 (1.01–1.16) | 0.018 |
| Dyslipidemia | 1.20 (1.10–1.31) | <0.001 |
| Chronic kidney disease | 0.96 (0.85–1.08) | 0.486 |
| Gouty arthritis | 0.86 (0.77–0.97) | 0.011 |
| Psoriasis arthritis | 1.13 (0.92–1.38) | 0.241 |
| Charlson Comorbidity Index score* | 1.15 (1.12–1.18) | <0.001 |
| Medication of primary interest* |  |  |
| PUVA or UVB | 0.74 (0.59–0.92) | 0.007 |
| Cyclosporine | 0.84 (0.48–1.45) | 0.523 |
| Oral retinoids (Acitretin) | 0.42 (0.28–0.63) | <0.001 |
| Tacrolimus | 0.18 (0.02–1.56) | 0.119 |
| Hydroxyurea | 2.50 (0.81–7.77) | 0.113 |
| Mycophenolate mofetil | 8.79 (3.26–23.69) | <0.001 |
| Azathioprine | 1.44 (0.82–2.54) | 0.208 |
| Methotrexate | 0.86 (0.69–1.07) | 0.185 |
| Etanercept | 3.10 (1.15–8.37) | 0.026 |
| Adalimumab | 4.27 (1.74–10.46) | 0.001 |
| Ustekinumab | NA | NA |
| Medication not of primary interest |  |  |
| Steroid (prednisone-equivalent dose)* |  |  |
| Never user | Reference |  |
| <5 mg/day | 1.04 (0.96–1.13) | 0.341 |
| ≥5 mg/day | 2.44 (2.22–2.68) | <0.001 |
| Statin* | 1.53 (1.38–1.70) | <0.001 |

* Time-varying covariates which may change in each person-quartile;

PUVA, photochemotherapy; UVB, ultraviolet B; HR, hazard ratio; CI, confidence interval; NA, not applicable.
